# The association between enteral nutrition with survival of critical patients with COVID‐19

**DOI:** 10.1002/iid3.1261

**Published:** 2024-05-08

**Authors:** Maryam Gholamalizadeh, Zahra Salimi, Khadijeh Abbasi Mobarakeh, Zahra Mahmoudi, Shirin Tajadod, Mahdi Mousavi Mele, Farkhondeh Alami, Bojlul Bahar, Saeid Doaei, Sara Khoshdooz, Masoume Rahvar, Somayeh Gholami, Masoume Pourtaleb

**Affiliations:** ^1^ Cancer Research Center Shahid Beheshti University of Medical Sciences Tehran Iran; ^2^ Student Research Committee, Faculty of Nutrition and Food Technology Shahid Beheshti University of Medical Sciences Tehran Iran; ^3^ Department of Community Nutrition, Nutrition and Food Security Research Center, School of Nutrition and Food Science Isfahan University of Medical Sciences Isfahan Iran; ^4^ Department of Nutrition Science and Research Branch Islamic Azad University Tehran Iran; ^5^ Department of Nutrition, School of Public Health, International Campus Iran University of Medical Sciences Tehran Iran; ^6^ Department of Nutrition University of Medical Sciences Tehran Iran; ^7^ Nutrition Sciences and Applied Food Safety Studies, Research Centre for Global Development, School of Sport and Health Sciences University of Central Lancashire Preston UK; ^8^ Department of Nutrition, Student Research Committee, Faculty of Medicine Urmia University of Medical Sciences Urmia Iran; ^9^ Razi Clinical Research Development Unit, Razi Hospital Guilan University of Medical Sciences Rasht Iran; ^10^ Intensive Care Unit (ICU), Razi Hospital Guilan University of Medical Sciences Rasht Iran

**Keywords:** COVID‐19, enteral nutrition, mortality, survival

## Abstract

**Background:**

Coronavirus disease 2019 (COVID‐19) results in several complications and mortality in intensive care unit (ICU) patients. Limited studies have investigated the effect of enteral nutrition (EN) on the survival of COVID‐19 patients in the ICU. The aim of this study was to investigate the association of EN with biochemical and pathological indices associated with mortality in ICU patients with COVID‐19.

**Methods:**

This case–control study was conducted on 240 patients with COVID‐19 hospitalized in the ICU including 120 eventual nonsurvived as the cases and 120 survived patients as the controls. All of the patients received EN as a high protein high volume or standard formula. Data on general information, anthropometric measurements, and the results of lab tests were collected.

**Results:**

The recovered patients received significantly more high protein (60.8% vs. 39.6%, *p* = .004) and high volume (61.6% vs. 42.3%, *p* = .005) formula compared to the nonsurvived group. Mortality was inversely associated with high volume (odds ratio [OR]: 0.45 confidence interval [CI]95%, *p* = .008) and high protein (OR: 0.42 CI95%, *p* = .003) formula. The results remained significant after adjusting for age and sex. Further adjustment for underlying diseases, smoking, body mass index, and the acute physiology and chronic health evaluation II (APACHE II) score did not change the results.

**Conclusion:**

The findings of the study showed that there was a significant inverse association between mortality and high volume and high protein formula in patients with COVID‐19. Further investigation is warranted.

## Graphical abstract

Characteristics of the coronavirus disease 2019 participants recovered and died after hospitalization intensive care unit.

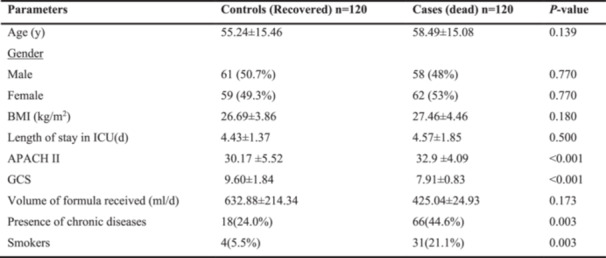

## 1 INTRODUCTION

Coronavirus disease 2019 (COVID‐19) is a relatively new respiratory disease that first appeared in Wuhan, China.^1^ The COVID‐19 disease has killed more than 5 million people globally ^2^ and has affected the lives of millions of people around the world due to the quarantine of cities, business closures, and social distancing.^3^ COVID‐19 is associated with many critical health issues such as severe acute respiratory syndrome.^4^ Since the outbreak of this contagious disease, the number of cases increased rapidly and it affects all age groups and people all over the world.^5^ Patients with COVID‐19 hospitalized in an intensive care unit (ICU) often need mechanical ventilation and extracorporeal membrane oxygenation, which increases treatment costs and complications.^6^ The financial crisis caused by this virus increased mental problems and suicide among people.^7^ The complications caused by this virus include pulmonary, cardiovascular, neuropsychiatric, hematologic, gastrointestinal, renal, endocrine, dermatologic, and musculoskeletal problems.^8^ However, the mechanism of the effects of factors causing long‐term complications of COVID‐19 is not yet clear.

There are several factors that affect COVID‐19 mortality such as age, lung disease, hypertension, heart disease, kidney disease, and metabolic disorders. In critically ill patients with Covid‐19, the body's metabolism may experience severe disturbances. For example, the function of pancreatic beta cells is often affected and the regulation of blood glucose is disturbed.^9^ Poor nutrition of critically ill patients may be associated with increased length of stay in the ICU and higher mortality.^10^ Due to the persistent pro‐inflammatory immune response, the risk of nutritional stress such as malnutrition is higher in patients with COVID‐19.

Enteral nutrition (EN) is the preferred feeding route in ICU patients to enhance the integrity of the gut and promote immune function.^11^ One meta‐analysis showed that mortality was lower in the early EN group compared with the delayed EN group.^12^ Another meta‐analysis study indicated that early EN is associated with a lower risk of mortality and SOFA (Sequential Organ Failure Assessment) score compared with delayed EN in critically ill COVID‐19 patients but it did not significantly (*p* > .05) reduce the length of hospital stay, length of ICU stay and days on mechanical ventilation compared to delayed EN or parenteral nutrition, respectively.^13^ However, some previous studies found contradictory results and reported that EN does not improve the status of COVID‐19 patients.^13^ The association between the amount and content of EN with mortality in ICU patients has been less investigated. So, the present study aimed to investigate the association of EN with biochemical and pathological indices associated with survival/mortality in COVID‐19 patients.

## 2 METHODS

### 2.1 Study design and participants

This case–control study was carried out from March 2021 to January 2022 on a total of 240 patients with COVID‐19 hospitalized in the ICU department of Razi Hospital, Rasht, Iran, All patients received gastric EN as administered by a specialist physician. Of these patients, 120 who eventually died after the study period were considered as the case group, and 120 age and sex‐matched patients who were discharged were considered as the control group. The required sample size was estimated according to a previous similar study^14^ using Open EPI online software.^15^ Exclusion criteria included patients who were younger than 18 years old, those who died for reasons other than COVID‐19, those whose medical records lacked the necessary information, and EN intolerance which was determined as the presence of persistent vomiting or a gastric residual volume (GRV) higher than 250 mL at any time resulting in forced interruption of EN. Some patients (*n* = 121) were assessed by a dietitian and received the required nutritional support containing high protein and high‐volume formula following the latest medical guidelines.^16^ Also, 119 patients hospitalized in the ICU department of the same hospital received the standard formula.

### 2.2 Data collection

General information on the patients including age, gender, height, weight, body mass index (BMI), length of stay in the ICU, the Acute Physiology and Chronic Health Evaluation (APACHE II) score, the Glasgow Coma Scale (GCS), volume of formula received, presence of chronic diseases, smoking, and survival were collected from the patients' medical record.

### 2.3 Statistical analysis

To compare the demographic, anthropometric, biochemical, and pathological indices of the two groups, independent *t*‐test and chi‐square tests were applied for quantitative and qualitative variables, respectively. The association between EN and mortality was investigated using logistic regression. The confounding factors including age, gender, BMI, length of stay in the ICU, the APACHE II, the GCS, presence of chronic diseases, and smoking were adjusted in various regression models. SPSS 21 (IBM Corp.) was used for all statistical analyzes considering the significance level of *p* < .05.

## 3 RESULTS

The general characteristics of the COVID‐19 patients included in this study are presented in Table 1. There was no significant difference between the two groups of patients (survived vs. nonsurvivors) for their age (55.24 ± 15.46 vs. 58.49 ± 15.08 years), BMI (26.69 ± 3.86 vs. 27.46 ± 4.46 kg/m^2^), and gender. However, the nonsurvived patients had a significantly higher APACHII, higher incidences of underlying disease and smoking compared to the control group (*p* < .01), and lower GCS compared to the control group (Both *p* < .001).

**Table 1:** Characteristics of the COVID‐19 participants recovered and died after hospitalization ICU.

Parameters	Controls (recovered) *n* = 120	Cases (dead) *n* = 120	*p*‐Value
Age (y)	55.24 ± 15.46	58.49 ± 15.08	.139
Gender			
Male	61 (50.7%)	58 (48%)	.770
Female	59 (49.3%)	62 (53%)	.770
BMI (kg/m^2^)	26.69 ± 3.86	27.46 ± 4.46	.180
Length of stay in ICU(d)	4.43 ± 1.37	4.57 ± 1.85	.500
APACH II	30.17 ± 5.52	32.9 ± 4.09	<.001
GCS	9.60 ± 1.84	7.91 ± 0.83	<.001
Volume of formula received (mL/d)	632.88 ± 214.34	425.04 ± 24.93	.173
Presence of chronic diseases	18 (24.0%)	66 (44.6%)	.003
Smokers	4 (5.5%)	31 (21.1%)	.003

Abbreviations: APACHE, Acute Physiology and Chronic Health Evaluation; BMI, body mass index; GCS, Glasgow coma scale.

The comparison of the volume and type of formula received among the case and control groups is presented in Table 2. The survived patients received significantly more high protein (60.8% vs. 39.6%, *p* = .004) and high volume (61.6% vs. vs. 42.3%, *p* = .005) formula compared to the nonsurvived patients.

**Table 2:** The comparison of the volume and type of formula received among the case and control groups.

	Controls (recovered) *n* = 120	Cases (dead) *n* = 120	*p‐*Value
Formula type			
Standard	47 (39.2%)	72 (60.4%)	.004
High protein	73 (60.8%)	48 (39.6%)	
Formula volume			
<600 mL/d	46 (38.4%)	69 (57.7%)	.005
≥600 mL/d	74 (61.6%)	51 (42.3%)	

**Figure 1:**
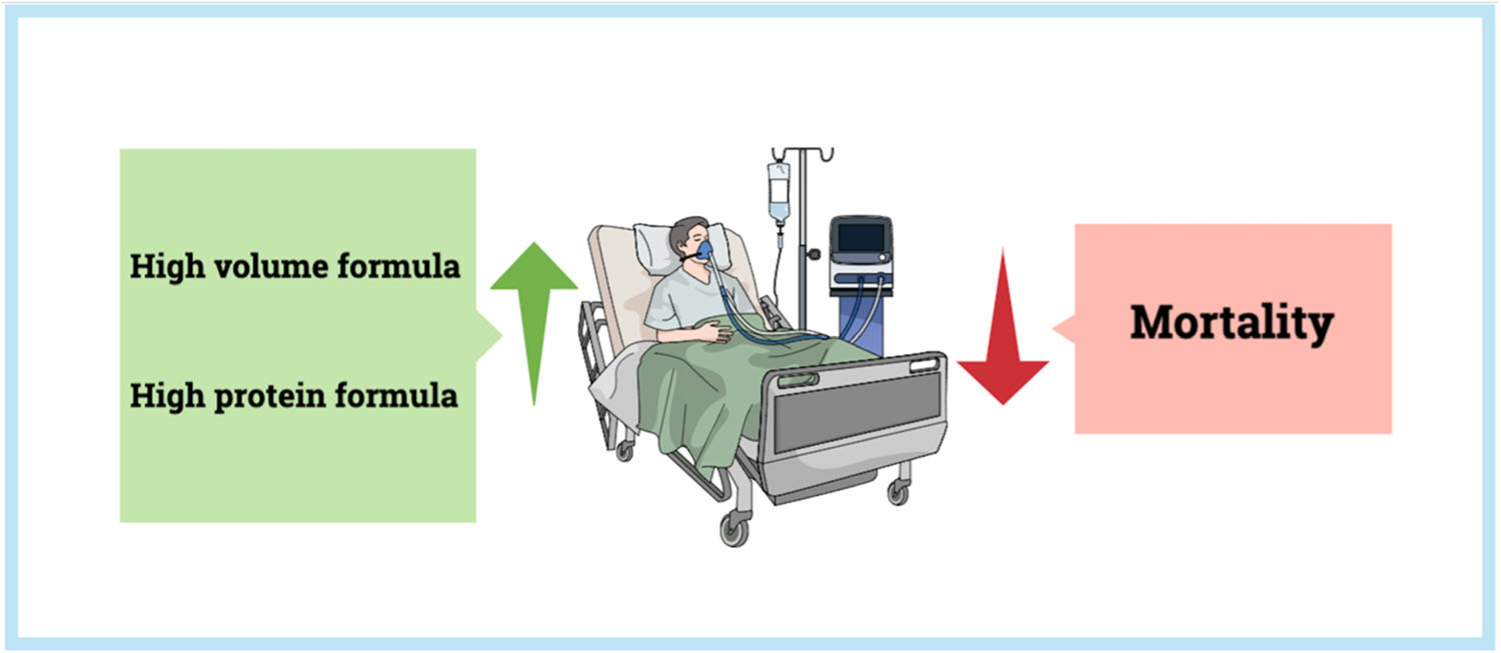
The effects of high‐protein and high‐volume formula on mortality in patients with COVID‐19.

The association between mortality with the volume and type of formula is presented in Table 3. Mortality was inversely associated with high volume (odds ratio [OR]: 0.45 confidence interval [C]I95%: 0.26–0.81, *p* = .008) and high protein formula (OR: 0.42 CI95%: 0.23–0.75, *p* = .003). The results did not change after adjustments for age and sex (model 2), further adjustments for underlying diseases and smoking (Model 3), further adjustments for BMI (Model 4), and additional adjustments for APACHII (Model 5) (Figure 1).

**Table 3:** The association between mortality with the volume and type of formula.

	High volume (≥600 mL/d)		High protein	
	OR (CI95%)	*p*	OR (CI95%)	*p*
Model 1	0.45 (0.26–0.81)	.008	0.42 (0.23–0.75)	.003
Model 2	0.42 (0.23–0.76)	.004	0.41 (0.23–0.74)	.003
Model 3	0.44 (0.24–0.82)	.010	0.43 (0.23–0.79)	.007
Model 4	0.445 (0.24–0.82)	.010	0.43 (0.23–0.80)	.008
Model 5	0.52 (0.27–0.97)	.043	0.44 (0.23–0.84)	.013

*Note*: Model 1: Crude, Model 2: adjusted for age and sex, Model 3: further adjustment for underlying diseases and smoking, Model 4: further adjustment for BMI, Model 5: further adjustment for APACHII.

Abbreviations: APACHE, Acute Physiology and Chronic Health Evaluation; CI, confidence interval; OR, odds ratio.

## 4 DISCUSSION

The present study investigated the association between EN and survival levels in patients with COVID‐19 admitted to the ICU. Our results indicated that the mortality was inversely associated with high volume and high protein EN after adjustments for age and sex, underlying diseases, smoking, BMI, and APACHII.

In line with this study, Martindale et al. recommend using a high‐protein (≥20% protein) polymeric iso‐osmotic enteral formula in the early acute phase of critical illness.^17^ Martindale et al.^17^ reported that EN should be initiated early after admission to the intensive care unit (ICU), while careful monitoring for gastrointestinal intolerance, hemodynamic instability, and metabolic derangements are essential.^18^ In addition, a recent retrospective study found that early EN in paralyzed patients was associated with decreased hospital mortality and no increase in ventilator‐associated pneumonia.^19^ Furthermore, in a recent Korean study, there was no significant difference in nutritional status or requirements at admission between survivors and nonsurvivors, but the actual intake of calories and protein was lower in the nonsurvivors group. Additionally, nonsurvivors had higher serum C‐reactive protein levels and lower serum albumin levels at discharge.^20^ Also, another study suggested that omega‐3 and arginine supplementation, in addition to meeting at least 80% of the energy and protein requirements, are associated with a reduced mortality rate among COVID‐19 patients.^21^ However, some studies reported contradictory results where no significant association between mortality and high protein formula was found.^22^ A possible reason may be that high protein formulas can improve the patient's condition only if they have a high volume and the calories needed by the patient are provided.

The exact mechanism of the effect of EN on the mortality of ICU patients with COVID‐19 is not clear. The beneficial effects of high protein formulas on the body's metabolism may be the cause of improving the patient's survival. Hyperglycemia when occurring in a critical physiological state^23^, ^24^ is likely to contribute to poor clinical outcomes.^25^ Insulin resistance and hyperglycemia result in muscle protein breakdown.^26^, ^27^ High‐protein nutrition helps glucose control, reduces insulin requirements, improves muscle synthesis, and provides substrates needed at the sites of tissue injury in critically ill patients.^28^, ^29^ The positive effect of protein is also attributable to the maintenance of nitrogen balance and lean body mass^30^ and its effect on the production of neurotransmitters, glutathione synthesis, and other compounds required during the acute infection phase.^31^ These results together strongly emphasize the importance of EN for survival levels in ICU patients with COVID‐19.

However, this research had some limitations. First, its observational design precludes the establishment of causation. The research was constrained to a single center, perhaps compromising the generalizability of the results. Also, some statistically adjusted factors such as chronic disease and smoking may influence the obtained results. Further studies are warranted to incorporate multicenter randomized controlled trials to validate the results and establish a causal relationship between EN and decreased mortality rates among patients inflicted with COVID‐19. Standardizing EN protocols including the timing, duration, quantity, quality, and monitoring of nutritional support should be a priority.

## 5 CONCLUSION

The present study provides the first evidence for an association between EN with survival of ICU patients with COVID‐19. According to the findings of the study, there was a significant inverse association between mortality and high‐volume and high‐protein formula. Prospective clinical trials and cohort studies should be performed to establish a causal relationship between EN and survival in ICU patients with different diseases and to discover the optimum quality and quantity of EN. If the results of the present study are confirmed in future research, it can be considered an effective strategy to reduce the mortality rate of these patients by recommending high‐calorie and high‐protein formulas to patients admitted to the ICU. Clinicians should adapt nutritional strategies accordingly, which could lead to updates in clinical nutrition guidelines for critical care patients. Future research should assess the influence of nutritional support on long‐term outcomes following discharge from the intensive care unit. Additionally, it is crucial to examine the potential impact of other influential factors, including genetic predispositions, comorbid conditions, and specific components found in enteral formulas.

## AUTHOR CONTRIBUTIONS

**Maryam Gholamalizadeh**: Data curation; investigation; project administration; supervision; writing—original draft; writing—review & editing. **Zahra Salimi**: Formal analysis; investigation; methodology; software; validation. **Khadijeh Abbasi Mobarakeh**: Investigation; resources; software. **Zahra Mahmoudi**: Investigation; project administration; writing—original draft; writing—review & editing. **Shirin Tajadod**: Conceptualization; data curation; software; validation; writing—original draft. **Mahdi Mousavi Mele**: Conceptualization; data curation; software; validation; writing—review & editing. **Farkhondeh Alami**: Investigation; project administration; software; validation. **Bojlul Bahar**: Formal analysis; methodology; software; writing—original draft; writing—review & editing. **Saeid Doaei**: Formal analysis; investigation; project administration; writing—original draft; writing—review & editing. **Sara Khoshdooz**: Visualization; writing—review & editing. **Masoume Rahvar**: Conceptualization; data curation; investigation; writing—original draft. **Somayeh Gholami**: Formal analysis; investigation. **Masoume Pourtaleb**: Data curation; formal analysis; investigation; validation.

## CONFLICT OF INTEREST STATEMENT

The authors declare no conflict of interest.

## ETHICS STATEMENT

This study was approved by the Institutional Review Board at Shahid‐Beheshti University of Medical Sciences (code: IR.SBMU.CRC.REC.1402.025.) All patients signed an informed consent form at baseline. Institutional consent forms were used in this study.

## Data Availability

The raw data supporting the conclusions of this article will be made available by the authors without undue reservation.
